# Supplementary material for: Are carbon emissions trading and green financial instruments synergistic? -Comprehensive quantitative research based on content analysis
Source: PLoS One. 2024 Mar 7;19(3):e0298601. doi: 10.1371/journal.pone.0298601 (PMC10919601; doi:10.1371/journal.pone.0298601)
Supplement: S1 Data — (ZIP) [file pone.0298601.s001.zip › Data/地级市Z_F工作报告.zip!/║ú─╧/║ú┐┌╩╨/└┤╫╘╣1⁄2╓┌║┼í╛┬φ┐╦╩2╛▌═°í┐_2013.docx]

各位代表：

　　现在，我代表市人民政府向大会报告工作，请予审议，并请各位政协委员和其他列席人员提出意见。

　　一、2012年工作回顾

　　2012年，面对复杂多变的宏观经济形势，在市委的正确领导下，我们以学习贯彻十八大精神为动力，深入落实科学发展观，凝心聚力，攻坚克难，较好地完成市十五届人大一次会议确定的年度目标任务，实现了经济平稳增长、社会和谐稳定。全年实现地区生产总值820.58亿元，增长9.4%；来自海口的公共财政收入210.43亿元，增长14.4%，其中地方公共财政收入73.17亿元，增长20.1%；固定资产投资510.38亿元，增长29.2%；社会消费品零售总额436.26亿元，增长12.7%；城镇居民人均可支配收入22331元，农村居民人均纯收入8134元，分别增长13.2%和13.1%；居民消费价格涨幅3.3%；城镇登记失业率1.32%；人口自然增长率8.18‰；全面完成省下达的节能减排任务。

　　（一）扩投资、上项目，科学发展更加持续。以前所未有的力度扎实开展“项目建设年”活动，固定资产投资增幅创1996年以来新高。90个省市重点项目完成投资320.18亿元，占年度计划的165.3%,可望获全省重点项目考核唯一的上台阶奖。红塔30万箱易地技改、英利二期400兆瓦、汉能250兆瓦、众联新能源电动球车等项目建成投产，龙昆南延长线、货运大道二期、绿色长廊二期、南海大道整治、羊山路网一期竣工通车，琼州文化风情街、水巷口骑楼示范样板街试运营，观澜湖旅游小镇、马村港二期、新海港滚装码头、永和花园百万平方米生态安居工程、南渡江防洪堤等项目加快推进，海榆中线拓宽、华谊冯小刚电影公社、生生国际购物中心、海垦商业中心、大印保税物流园等项目启动建设，美兰机场二期扩建批准立项。

　　（二）调结构、兴产业，发展方式加快转变。坚持把发展实体经济，培育发展能够带来可持续税收、增加就业、具有支柱引领作用的特色优势产业作为经济结构调整的重中之重。提升农业比较效益，完成增加值57.74亿元，增长6.2%。新建常年蔬菜基地1.06万亩、花卉4000亩、设施大棚1950亩、标准化养殖小区15个、30万只蛋鸡养殖场2个，新成立农民专业合作社242个，罗牛山农产品加工产业园3个子项目主体工程封顶。30万亩土地整治项目已到位国家和省支持资金8.5亿元，开工建设10个项目，整治土地6万亩，年度完成投资6.48亿元，其中马坡洋万亩蔬菜基地、柏盈兰花产业园实现当年建设、当年供菜出花。做大工业总量规模，全市工业完成总产值561.29亿元、增加值136.67亿元，分别增长8.3%、9.1%，其中高新区工业产值增长46%。投入4.12亿元，完成园区基础设施项目建设17个，美安科技新城总体规划、一期控规和产业规划通过评审，一期工程开工建设。加大科技创新和企业技术研发扶持力度，争取国家和省科技创新资金7400多万元，全年新增国家级高新技术企业22家、省级工程技术研发中心3家和省级重点实验室8家。海口国家绿色科技产业国际创新园获科技部批准。加快第三产业转型发展，完成增加值561.17亿元，增长9.4%。积极落实离岛免税新政策，开展“城市营销年”活动，组织各类岛外专项促销10多场次，举办第二届“海口之春”旅游艺术节、观澜湖世界明星高尔夫邀请赛等文化赛事活动30多个，全年接待国内外过夜游客952.9万人次，旅游总收入完成101.57亿元，分别增长12.7%、22.3%。改变了旅游业长期南热北冷的格局，实现了旅游业回升向好的重大转折，旅游服务满意度位居全国前列。成立会展局，商务会展突破200场次，荣获2011-2012年度中国品牌会展城市大奖。启动海口会展购物中心项目，观澜湖兰桂坊大型商业项目主体工程封顶，开工建设综合保税区公共仓储物流基地，海口港集装箱年吞吐量突破百万标箱，正式跨入国内集装箱大港行列。认真兑现金融扶持政策，中信、招商等银行海口分行和海口农商银行挂牌运营，双成药业成功上市，海南港航企业债券上市发行，金融业增加值增长18.5%。积极调整房地产结构，商业及办公设施规划报建占比达到25.9%；销售商品房266万平方米，增长18%。

　　（三）配资源、优服务，调控能力明显提高。积极发挥政策导向作用，先后制定出台加快工业、会展、民营经济和总部经济发展等扶持政策，投入产业发展资金4.13亿元，其中对157家工业企业兑现政策奖励扶持资金1.35亿元，为中小企业争取国家、省级扶持资金5100万元和商贸企业发展资金1.62亿元，支持中小企业担保贷款22.59亿元；对英利新上1G瓦项目、观澜湖旅游小镇、海马第三工厂等重点项目实行差别化政策，配置优质资源，提供保姆式服务；增加航空、旅行社奖励资金8000万元和1200万元。对10家医药骨干企业实行“一企一策”，帮助企业依法用足税前扣除政策，解决人才引进、生活配套等问题。积极争取省政府免除工业发展资金及占用费5亿元，争取省级地方债券专项补助资金3.3亿元。争取省市财政管理体制调整，共享税市级分享比例从45%提高到55%。

　　坚决稳妥有序开展闲置土地清查，基本摸清全市建设用地情况，进一步规范土地开发市场秩序，有效促进了项目加快开发和社会投资增长。

　　（四）推改革、促开放，发展活力进一步释放。全力以赴申报国家统筹城乡综合配套改革试验区，总体方案已上报国家发改委。认真落实市委《关于进一步深化行政审批制度改革的决定》，出台12个行政审批规范性文件，全面归并审批职能，实施联审联办、全程代办，行政审批速度提高40%，被评为2012年全国“服务型政府十佳城市”第六名。探索推行网络问政，邀请市民代表列席政府常务会议，行政改革举措得到《人民日报》、《光明日报》等众多媒体重点报道。采取BT模式融资启动秀英港整体搬迁、海秀快速路等重大基础设施建设，实行“限地价、竞房价”的新型融资模式进行保障性住房建设。落实国土资源部支持海南国际旅游岛建设20条政策，制定25条实施意见。加快镇域经济发展，启动计划单列镇改革，创新城市社区管理，8个社区开展网格化服务试点。桂林洋农场属地管理承接工作顺利完成。积极扩大对外开放，全年举办大型专题招商推介会16场，先后引进中投、中交、中铁等大企业入驻。保障第八届泛珠大会成功举办，签约10个重大项目、总投资1205亿元。创立琼北“7+1”旅游区域合作联盟，成功举办省会经济圈2012年度论坛、华商圆桌会议。全年实际利用外资4.5亿美元，增长11%；出口总额18亿美元，增长6.2%。

　　（五）强基础、拓功能，城乡面貌明显提升。扎实开展“科学规划年”活动，完成新一轮城市总体规划和土地利用总体规划修编，以及6个片区控规、17个专项规划，主城区控规实现全覆盖；在全省率先开展村庄规划，完成主城区外17个镇总规、131个行政村村域规划和1196个自然村建设规划的编制。片区开发和棚户区改造步伐加快，金沙湾片区、西海岸南片区一期市政工程顺利开工，美丽沙、新埠岛、大英山片区开发初具规模，南海明珠、如意岛等填海工程和司马坡文体基地加快推进。创新改造模式，上贤村、夏瑶村棚户区改造实现盈利或平衡。丘浚休闲广场开工建设，海瑞文化园完成征地拆迁协议签订97.8%，五公祠改造进展顺利。“9+1”市政道路贯通工程按计划通车，新增调整公交线路35条，投放运营节能与新能源公交车577辆，投放更新出租车280辆，开工建设公交场站7个。深化道路交通专项整治，优化国贸片区、海秀中路片区等交通组织，完成电动自行车登记上牌32万辆。积极创建 “全国文明城市”、“国家卫生城市”，推进“五大工程”环境综合整治，全年共拆除违法建筑663宗、83.12万平方米。扎实开展“绿化宝岛”大行动，造林3.37万亩，启动永庄水库、沙坡水库、玉龙泉3个森林公园和白水塘湿地公园前期工作。颜春岭生活垃圾焚烧发电厂建成运营。新建农村家庭卫生厕所8000户。在全省率先公布PM2.5实时监测数据，环境空气质量优良率达到100%，顺利通过“国家环境保护模范城市”现场复核。

　　（六）惠民生、促和谐，人民生活显著改善。在财政收入持续快速增长，可望获全省市县财政收入规模上台阶奖第一名的基础上，全年民生支出79.51亿元、增长16.2%，占地方公共财政支出的70.1%。省政府部署的8件实事和市委、市政府承诺的10件实事全部完成。稳定和扩大就业，扶持完善创业孵化基地23家，发放创业小额贷款4664万元，全年城镇新增就业人口5.1万人、增长28%，农村富余劳动力转移就业1.35万人，零就业家庭动态清零，被国务院评为首批“全国创业先进城市”。不断提高社会保障水平，最低工资标准提高到1050元，企业退休人员基本养老金月人均增加289元，新农保养老金月标准提高到85元，城镇、农村低保月标准分别提高到390元和300元，农村“五保”供养月标准提高到350元，发放高龄老人长寿补贴4260万元。完成乡村敬老院建设4所，新增床位530张。城镇从业人员医保、城镇居民医保报销封顶线分别提高到26万元和12万元，城镇居民医保重大疾病报销封顶线提高到22万元，发放城乡医疗救助金1639万元。新建保障性住房15656套，对3346户家庭发放廉租房补贴；完成农村危房和水库移民危房改造3284户，农村危房改造工作荣获省一等奖。集中开展已售住房未办证清理专项行动，为已售住房办证18042户。积极推进义务教育均衡发展，加快推进校安工程建设，302个校安工程已完工278个、竣工率达92.1%，启动公办幼儿园建设16所，在全省率先发放学前教育助学券，实施公开透明的“阳光招生”政策。丰富城乡群众文化生活，开展各类文化体育活动4000多场次，组织文化下乡36场。提高基层卫生服务水平，新建农村标准化卫生室64家，全市农村标准化卫生室达标率100%，城镇居民规范化电子档案建档率达75%，免费为城乡居民提供11项基本公共卫生服务和5项重大公共卫生服务。人口和计生综合改革工作取得新进展，再次被评为全省人口和计划生育工作先进市。积极维持物价稳定，调动1.05亿元价格调节基金对猪肉和蔬菜价格、公交公益性服务等实行补贴，升级改造农贸市场27家，新增农副产品平价商店25家。强化基层基础建设，构建“大调解”新机制，化解各类矛盾纠纷7839宗，办理法律援助案件6615宗，全市信访总量同比下降19.9%。开展打击“两抢”、“破案会战”、“禁毒斗争”等专项整治行动，社会治安形势稳定。严格落实安全生产责任制，全年未发生重特大事故。实现全国双拥模范城“七连冠”。食品药品、粮食、民防、统计、气象、档案、史志、外事侨务、民族宗教等工作取得新成绩，工会、妇女、儿童、青少年、老龄、慈善、残疾人等事业取得新进步。

　　（七）转作风、提效能，政府自身建设不断加强。认真执行市人大及其常委会的决议，主动接受市人大、市政协监督，办理人大代表建议429件，政协委员提案385件，办结率均达100%。扎实开展“企业走访月”活动，为企业解决26大类、100多个实际难题。完善重大事项集体决策、专家咨询、社会公示和听证制度，加强政务公开，推进电子政务，行政复议工作获全国表彰，依法行政工作全省考核第一名。强化行政监察和审计监督，深入开展集中整治“庸懒散奢贪”、工程建设领域突出问题、公务用车和“小金库”等专项治理，纠正部门和行业不正之风，廉政建设有力推进，政府执行力和公信力进一步提升。

　　各位代表！过去一年，我们取得的成绩来之不易，这是在省委、省政府和市委的正确领导下，全市人民团结奋斗的结果。在此，我代表市人民政府，向全市人民致以崇高的敬意！向人大代表、政协委员，向各民主党派、工商联、各人民团体和各界人士，向驻市部队、武警官兵和公安民警，表示衷心的感谢！

　　在肯定成绩的同时，我们更应该清醒地看到，经济社会发展还存在不少困难和问题：一是经济增速受严峻宏观形势影响明显放缓，地区生产总值增幅等主要指标虽然高于全国全省平均水平，但没有实现年初预期目标，地方公共财政收入增幅相对缓慢，主要是因为产业结构单一，工业实体发展不够，现代服务业发展不足，经济运行存在较大潜在风险。二是城市建设管理还存在薄弱环节，社区管理服务滞后，城区违规建房等问题还需从源头加强治理。三是政府部门工作人员的思想观念、服务意识、工作作风、办事效率和执行能力尚不适应工作需要，整体素质有待进一步提高。对此，我们必须认真对待，妥善解决。

　　二、2013年的主要工作任务

　　坚持科学发展、实现绿色崛起，今年的工作至关重要。政府工作的总体思路是：全面贯彻党的十八大精神，以科学发展观为指导，认真落实省第六次党代会和市第十二次党代会各项部署，把保持经济平稳健康发展作为首要任务，把建设海南“首善之城”作为主要目标，进一步提高经济增长质量和效益，扩大有效需求，做强优势产业，统筹城乡发展，创新体制机制，保障改善民生，努力开创“宜居、宜业、宜学、宜游”和“养眼、养身、养心”最精最美省会城市建设新局面。

　　综合考虑各方面因素，今年我市经济社会发展的主要预期目标是：地区生产总值增长10%左右；地方公共财政预算收入增长15%；固定资产投资增长23%；社会消费品零售总额增长15%；城市居民人均可支配收入增长10%；农村居民人均纯收入增长11%；居民消费价格涨幅控制在5%以内；城镇登记失业率控制在3%以内；人口自然增长率控制在10.2‰以内；全面完成省下达的节能环保指标。

　　重点抓好以下六个方面的工作：

　　（一）千方百计扩大有效需求，保持经济平稳较快增长

　　加大项目建设年推进力度。安排市重点项目75个，力争固定资产投资突破600亿元。启动长影“环球100”、海南电影公社、火山群国家地质公园一期、海南野生动植物园新区、海文高速公路改造等项目建设，加快推进马村港中心港区、新海港一期、海秀快速路、华谊冯小刚电影公社、东寨港红树林生态旅游片区开发保护、观澜湖旅游小镇、海航日月广场、永和花园百万平方米生态安居工程、骑楼历史文化街区保护和重点人文景点升级改造等工程建设，完成秀英港散件码头搬迁。落实责任，强化措施，确保项目早竣工、早投产、早见效。

　　大力引进和扩大消费。巩固和扩大岛外消费市场，积极培育促销主体，力争全年旅游、房地产、农产品、会展赛事等大型岛外促销活动达到30场次、规模以上会展活动超过220场次；接待国内外过夜游客突破1100万人次，旅游总收入超过113亿元、增长13%；完成商品房销售320万平方米、增长21%。放大离岛免税政策效应，完成观澜湖免税店建设；引进2-3家商业龙头企业，扶持发展新型商业模式，着力开发与节假日调整相适应的热点消费，提升消费层次。深化“万村千乡市场工程”和“新网工程”，加快鲜活农产品直供直销体系建设，改造农贸市场6家，新建农家店20家，扩大农村消费需求。努力增加城乡居民特别是中低收入者收入，建立完善工资正常增长机制和支付保障机制，落实社会最低工资标准，扩大城乡低保覆盖面，加大价格补贴力度，带动消费增长。力争社会消费品零售总额超过500亿元。

　　加强经济运行调节。抓住国家实施积极财政政策，积极争取中央、省预算内资金，力争我市专项转移支付资金全省占比达到18%以上。做好营业税改增值税前期准备，全面开展涉企行政事业性收费清理，建立“涉企收费明白卡”制度，增强企业投资意愿和能力。加强银企对接，引导金融机构加大对基础设施、“三农”、优势产业、重点项目和小微企业的信贷投放，力争全年信贷投放规模增长20%以上。鼓励企业上市融资。用活产业扶持发展、科技兴工、企业技改、担保贴息等各类资金，提高财政资金使用效益。发挥好土地杠杆调控作用，实行用地指标动态调整，保障重点项目和新引进企业用地需求。

　　（二）加快发展特色优势产业，进一步优化经济结构

　　推进工业提速提质。坚持“实业兴市”，把工业优先放在更加突出的位置，着力改造提升汽车制造、生物医药、食品饮料及农副产品加工、机电制造、化纤纺织及光伏产业等六大支柱产业，加快培育发展新能源、新材料、旅游装备、电子信息、节能环保和文化创意等六大新兴产业。加快园区平台建设，全面启动美安科技新城一期基础设施建设，建成云龙产业园污水处理厂。加快英利1G瓦、先声药业产业园、立升超滤膜、海马第三工厂、海宇20万吨镀锡原板扩建、大印保税物流园、高档消费品加工交易等项目建设，确保皇隆制药等一批医药企业竣工投产，力争高新区、综合保税区工业总产值分别增长20%和13%以上。大力实施“骨干企业壮大工程”、“中小企业成长工程”，支持英利、椰树、金盘电气、齐鲁制药、双成制药等优势骨干企业做大做强，力争新增规模以上工业企业4家。加快以企业为主体的科技创新体系建设，支持有条件的企业建立技术研发中心，形成核心技术和主导产品，力争新增国家认定高新技术企业10家，发明专利授权量增长5%以上。鼓励企业积极创建名牌产品或著名商标，争创省著名商标12个、名牌产品8个。完善适应中小企业特点的担保融资体系，为各类企业提供担保贷款增长15%以上。全面完成省政府下达的节能减排任务。力争全年工业增加值突破150亿元，增长12%以上。

　　加快服务业提档升级。积极申报国家现代服务业综合试点。立足特色，大力发展文化旅游、商业旅游和乡村旅游。深化琼北旅游合作，创新旅游营销体系，推进旅游市场和客源结构多元化，推出“智慧旅游—乐旅海口”、“琼北过大年”等活动，创建全国旅游标准化试点城市。建立海口区域文化产业项目数据库，以海南国际创意港为依托，全力推进海南广告产业园建设，加快文化创意产业发展。继续扩大“海口之春”、观澜湖高尔夫世界杯等重大文体旅游节庆活动品牌效应。围绕构建区域性商贸物流中心，加快海秀东、国贸、大英山城市商圈建设，推动海口会展中心购物城、生生国际购物中心易地扩建、海垦商业中心、海口现代美居生活园建设，加快美安物流园、美兰国际空港物流中心、马村港保税物流中心建设。抓住“建设海洋强省”的战略契机，利用海口在海洋行业、海洋科技等方面的基础优势，积极引进海洋开发企业落户海口，为南海资源开发和三沙市建设提供全方位的技术支撑和后勤保障。加快建设环北部湾金融中心，推进海口城市商业银行筹建，确保浦发银行海口分行、华夏银行海口分行和海口联合农商银行开业运营，吸引3—5个区域性企业研发总部、结算总部、培训总部、航运总部和商贸物流总部落户。继续调整优化房地产开发结构，提高高端旅游、商业地产占比，促进房地产业健康发展。大力推进房产信息化建设，进一步解决房产“办证难”问题。提升餐饮娱乐等传统服务业，加大养老休闲、养生保健、社区服务、电子商务等新兴服务市场开发力度，发展会计、法律、咨询、评估等中介服务业，提高现代服务业发展水平。力争第三产业增加值增长10%以上。

　　增强热带特色现代农业竞争力。完成南渡江30万亩土地整治一期工程，启动二期工程，实施好田间工程、高效农业示范工程等4个子项目和15万亩土地整治。加快农业产业结构调整，制定农业产业发展规划和扶持政策，加快推进国家现代农业示范区建设，吸引更多的社会资金投向农业，重点抓好5个规模化、标准化、产业化的“菜篮子”片区、3个特色畜牧生产区、2个花卉产业园和1条花卉长廊建设，新增1000亩设施大棚、10个标准化冬季瓜菜基地和20个标准化养殖小区。积极培育无公害、绿色、有机农产品品牌，重点打造5个富硒、富锗知名品牌。加快国家级水产物流交易中心建设。支持罗牛山等农业龙头企业加快改制改造，开发新产品，延伸产业链，提升农业附加值。新增省级农业产业化龙头企业3家。加强农业科技服务体系建设，健全农业科技推广、动植物疫情防控、农产品质量监督等公共服务机构。提高农业组织化水平，新增农民专业合作社30家。力争农业增加值增长7%以上。

　　（三）积极稳妥推进城镇化，统筹城乡均衡发展

　　做大做精主城区。继续提升城市畅通能力，以申报“公交都市”建设示范工程第二批创建城市为目标，抓紧筹划BRT快速公交，推进现代有轨电车一号线一期工程前期工作。加快公共设施建设，新建改建一批街边街心绿地，启动五源河文化体育中心和国贸、海秀、府城等核心商业区配套设施建设，加快城市排涝体系和排水管网建设，启动椰树门广场人防工程及体育场改造，完成白沙门（双拥）公园改造、万绿园地震应急避难所建设。启动“无线海口”建设，打造便民服务信息化平台。抓好已建保障性住房小区市政配套建设。继续推进旧城改造和片区开发，加快推进国贸椰岛片区、国贸一横路片区、流水坡片区棚户区改造，启动白沙坊、盐灶、博义等棚户区改造，加快西海岸南片区、金沙湾、大英山、江东、新埠岛等片区开发和如意岛、南海明珠二期等填海项目建设，确保完成年度投资计划。

　　做强做特中心镇。认真落实《关于加快镇域经济发展的决定》、《关于对云龙等镇实施计划单列试点的意见》，加大镇级专项转移支付，统筹安排镇域基础设施建设项目，(删除此句：采取提高市政基础设施配套费、按一定比例返还土地出让金等方式，)增强中心镇自主发展能力。加快镇域规划编制，配备村镇发展规划人员。启动江东大道、永兴至三江贯通工程，改造县道68.2公里。加快农村电网升级改造。推进云龙工贸综合服务镇、红旗花卉产业示范镇、演丰乡村旅游镇、石山火山旅游休闲镇（添加此句）、永兴旅游风情镇、新坡休闲农业观光镇等中心镇、特色镇建设，下大力气培育发展“一镇一品”、“一镇一业”，推进二、三产业向镇墟发展，提升中心镇产业承载力。加快农业资源与生态品牌、自然景观等资源禀赋的结合，引进社会资本，打造生态果园、花卉长廊、乡村酒店等一批特色农业旅游项目，逐步建设百里百乡生态休闲旅游网，年内新建15家休闲农业示范基地。

　　做优做美新农村。强化村庄规划，抓紧建设一批新型农村社区。统筹规划，捆绑项目，整合资金，加大农村基础设施建设投入，继续改善农村生产生活条件，启动新坡至东山段排涝、龙泉至新坡段防洪、荣山河防洪治理和演丰、三江、桂林洋防潮堤工程，争取南渡江引水工程获国家立项批复。加快推进农村土地流转，促进土地向种养大户集中，向龙头企业集中，向合作组织集中，提升农村土地规模效益。继续开展“阳光工程”、“雨露计划”，完成农民产业技能、务工技能培训5000人次，培养一批科技致富带头人。继续落实强农惠农富农各项政策措施，制定和实施全市农民收入倍增计划。

　　做深做实生态文明。加强对大气、水源、土壤和重金属污染防治力度。重点整治机动车排气污染和建筑施工扬尘污染，全面实施机动车环保合格标志管理，确保我市大气环境质量保持一流水平。强化饮用水水源地环境综合整治，持续改善城区水环境质量，加快（将“完成”改为“加快”）白沙门污水处理厂一期除磷脱氮升级改造和龙塘污水处理厂管网建设，保障饮用水质量安全。继续加大生态循环农业建设，新建沼气工程项目17个，户用沼气池500个，全市生态循环农业覆盖面积达到12万亩。加大旅游名村、生态文明示范村创建力度，推进“绿化宝岛”大行动，完成植树造林2.13万亩。加大环保执法监督力度，加强生态文明教育，增强全社会生态文明意识。

　　（四）深入推进改革开放，增创体制机制新优势

　　继续深化各项改革。以争取国家统筹城乡综合配套改革试验区为总抓手，加快农村土地制度改革，建立农村建设用地交易市场，开展农村土地流转试点(删除此句：；在云龙、红旗、龙泉等镇和江东片区实施土地管理创新制度试点，逐步实行城乡建设用地“同地同价”)。着手制定户籍制度改革方案。加快政府职能转变，继续深化行政审批制度改革，精简规范审批事项。创新社会服务管理，加快培育发展社会组织，建立公益性事业“以钱养事”、政府购买公共服务的新机制。完善市区财政体制，全面启动城市社区管理体制改革，下沉资源、项目、事权，建立标准化基层公共服务平台。深化政府投融资体制改革，完善城市基础设施BT、BOT、EPC建设模式，搭建农田水利融资新平台，加快（将“完成”改为“加快”）第二水务对外招商；健全政府投资项目可研及概算审核制度、概算定额指标制度。进一步整合职能，深化“大城管”、“大交通”城市综合管理改革。稳步推进事业单位改革。积极推进劳教制度改革。继续推进公立医院改革试点工作。

　　积极主动扩大开放。切实提高对外开放的质量和水平，强化招商引资目标考核，推行重大招商项目审批全程代办制，力争全年招商引资增长12%以上，其中实际利用外资超5亿美元，增长13%。落实柔性引进人才计划，对高素质人才落地创业，在户口迁入、职称评定、子女入学、住房保障和个人所得税等方面给予优惠，重点引进一批我市经济转型升级和发展方式转变急缺的高端领军人才，构筑加快发展的智力支撑体系。深化省会经济圈在规划、重大基础设施建设、产业布局、公共服务配套等方面的工作衔接，进一步融入泛珠三角和环北部湾区域。办好第三届中国南方（海口）国际合唱艺术周、第十届中国国际物流节等一批重大文化赛事会展活动，争取赢得2014年世界城市和地方政府联合组织理事大会、2017年世界石油大会举办权。制定外贸出口企业发展扶持政策，力争外贸出口增长13%。

　　（五）把管理放在更加重要的位置，努力建设全省首善之城

　　注重规划引导调控。深入开展“科学规划年”活动，强化规划的要素聚集功能，增强规划的科学性。按照现代宜居城市建设要求，进一步完善城市总体规划，统筹各类规划的编制，优化城市色彩、城市轮廓线、标志性建筑设计，对新建区域实施规划控制，对建成区实施规划改造，高水平设计一批街区和单体建筑风貌样式，彰显城市特色。继续推进规划向农村延伸，完成所有镇的总体规划、行政村村域规划和自然村建设规划编制，逐步形成覆盖城乡的规划体系。加大规划实施的监督执行力度，保持规划的科学性、前瞻性、系统性、权威性、严肃性，坚决防止规划的随意调整。构建城乡一体的规划国土管理新体制，继续加大闲置土地清查力度，科学合理安排建设项目用地，确保省市重点项目落地建设。

　　强化城市精细管理。整合规划、建设、城管、公安、交管、环卫、环保、民政等部门信息资源，建成城市综合管理数字化指挥监督系统。提请市人大常委会审议《海口市城市综合管理条例》，建立城管问题及时发现、及时处理和督查反馈机制，提升城市管理法治化水平。整合力量，综合施策，建立源头“打违”长效机制，坚决制止新的违建行为。加大疏导保畅力度，优化道路交通组织，对主要拥堵路段及学校、医院、大型商场等重要节点加强交通疏导，综合整治违禁行驶、乱行乱靠、横穿马路等交通违规行为。推进市容环境专项治理，全力整治城乡结合部卫生环境及市场摊区、露天烧烤、基建工地、泥头车等卫生污染源，努力让百姓生活的城市更干净、更整洁、更有序、更舒适。

　　加强和创新社会管理。加大社会管理创新力度，建立健全群众权益维护、公共服务管理、社会组织发展、公共安全保障、信息网络管理、社会稳定风险评估等工作机制，提高应急管理能力。加大对孤寡老残和精神病人、留守流浪儿童、服刑人员未成年子女等特殊人群的关爱帮扶，帮助他们解决生活实际困难。加大矛盾纠纷排查化解力度，及时处置苗头性、倾向性、突发性问题，防范社会风险。进一步强化信访工作责任，规范信访秩序，依法依规解决好群众反映的热点难点问题。加强精神文明建设，强化社会公德教育和诚信教育。深入推进“法治海口”、“平安海口”建设，加强社会管理综合治理，完善立体化社会治安防控体系。全面推进安全生产标准化建设，继续加大“打非治违”和隐患排查治理力度，坚决遏制重特大安全生产事故发生。深入推进“双拥”共建，促进军政军民团结。

　　不断提升城市文明。深入推进“全国文明城市”、“国家卫生城市”创建，广泛开展“讲文明、除陋习、树新风”素质教育活动，切实抓好文明村镇、文明社区、文明家庭创评工作，集中治理有损城市形象的不文明行为，培养市民主人翁意识和法规意识，进一步调动广大市民参与城市管理的积极性和主动性，共建文明温馨家园。

　　（六）切实保障和改善民生，努力实现群众美好生活新期待

　　提高社会保障水平。继续提高新农保基础养老金待遇，提高城市低保、农村五保供养标准和新农合、城镇居民医保参保人员财政补助标准，积极推进被征地农民养老保险工作，探索老农保、新农保、城镇居民养老保险和被征地农民养老保险等4项险种制度的整合，探索城镇居民医保和新农合医疗制度并轨，确保城乡居民养老保险参保率达95%以上，基础养老金发放率100%，确保基本医疗保险覆盖人群稳定在95%以上。新建4所乡村敬老院，城区每个社区建立1家居家养老服务站。按新的扶贫标准实现2130人脱贫。完善保障性住房分配和管理制度，真正惠及低收入困难群众。

　　努力保持物价总水平基本稳定。加强价格监管，严肃查处恶意炒作、哄抬物价等不法行为。以确保食品药品安全为前提，抓好烟草、酒类、食品、药品、农资、粮油、废旧品回收等市场专项整治，建立质量标准追溯体系，严厉打击制假售假行为。狠抓“菜篮子”工程，新建8330亩常年蔬菜基地，新设10家农副产品平价商店，主城区农贸市场设立平价公益摊位，确保不低于30%的价格调节基金对蔬菜、肉蛋等农副产品生产及价格进行适时调控。继续实施物价联动补贴制度，确保困难群众基本生活。

　　提升公共服务水平。加大资金投入，整合教育资源，整合“麻雀学校”，改善办学条件，强化师资力量，统筹推进各类教育协调发展，年内新改扩建幼儿园14所，在青少年宫原址新建1所九年一贯制学校，创建2所省级、5所市级义务教育规范化学校，确保所有校安工程年内竣工并交付使用。试点试行“先看病，后交费”制度，提升基层卫生医疗服务水平，巩固和完善全市基层医疗卫生机构实施国家基本药物制度成果，免费为城乡居民提供基本公共卫生服务和重大公共卫生服务项目，力争社区卫生服务覆盖率达100%，城乡居民规范化电子健康档案建档率达80%。千方百计稳定低生育水平，全面实施国家免费孕前健康检查，切实加大出生人口性别比综合治理，统筹解决人口问题。丰富基层文化生活，举办城乡各类公共文化体育活动4000场次以上，为农民放电影3000场以上，完成12个农村行政村文化室建设。继续做好编制、外事、侨务、民防、民族宗教、气象、档案、邮政、通信、史志、老龄、残疾人等工作。

　　各位代表，为群众办实事、谋福利是政府义不容辞的责任。今年，市政府将按照“尽力而为、量力而行，保基本、可持续”的原则，继续办好8个方面20件民生实事。一是实施交通畅通工程，继续打通“断头路”，新建金牛路北段、金垦路延长线、金宇东路、海垦中路等11条道路和海大北门人行天桥；扩大公交覆盖面，新开辟公交线路4条，新建候车亭100个以上，更新投放环保公交车100辆以上；优化滨海大道、海秀东路、国兴大道、长堤路等道路交通组织，增设10套人性化横道线红绿灯系统。二是实施文化惠民工程，开工建设白水塘湿地公园、沙坡水库森林公园；修缮秀英炮台，恢复对外开放；建设世纪公园市民公共文化广场和10个街心小游园。三是实施卫生改善工程，完成旧州、谭文等2个农村卫生院改造；启动水质检测体系一期建设，开展饮用水106项指标检测工作。四是实施教育提升工程，新建4所公办乡镇中心幼儿园；义务教育阶段学生免费乘坐公交车对象扩大至初中生。五是实施村镇社会事业工程，推进农村垃圾收集运转体系建设，建设43个行政村生活垃圾收运工程；推进广播电视邮政“村村通”建设，新建广播站300个，新增农村电视用户1.75万户，完成107个村邮站建设。六是实施居民副食品价格稳定工程，适当提高低收入群体和特殊群众物价补贴；各区新建1个小型公益性蔬菜直销市场。七是实施居民安居工程，建设保障性住房9900套，完成农村危房改造3500户；调整保障性住房收入标准，降低准入门槛，提高配售透明度，扩大保障覆盖面；将廉租房货币补贴标准提高至月人均每平方米18元；加快房产办证速度，解决40万平方米的房产证历史遗留问题；推进新建住宅小区用电用水抄表收费到户。八是实施就业促进工程，组织职业技能和创业培训1.2万人次，城镇新增就业4.2万人次。

　　各位代表！做好今年工作，必须切实加强政府自身建设，推进政府管理创新，努力建设为民务实清廉政府。一是全面提高依法行政和科学决策水平。严格按照法定权限和程序行使权力、履行职责、规范行为。自觉接受市人大及其常委会的法律监督和工作监督，主动接受市政协民主监督。认真执行市人大及其常委会的决议，坚持重大事项、依法行政情况报告制度，提高人大议案、代表建议和政协提案的办理质量。真诚接受民主党派、工商联和无党派人士的监督。坚持重大事项集体讨论决定，完善公众参与、专家论证和政府决策相结合的机制，健全社会听证制度。对关系群众切身利益的重大事项和重点项目，健全风险评估机制。二是进一步优化发展环境。深入开展“投资发展环境整治”活动，对行政审批过程中拒不办理、超时限办理、增设前置条件、强行有偿服务、相互推诿扯皮以及吃拿卡要等违纪违法的，有一起坚决查处一起；对乱检查、乱罚款、乱收费、乱摊派等损害市场主体利益行为的，有一起坚决查处一起；对企业发展、项目建设中出现各种困难矛盾、需要急事急办、特事特办、开通绿色通道的，有一件及时依法办理一件。严格执行各项扶持和促进发展的政策措施，大力营造良好发展环境。三是进一步转变工作作风。认真落实关于改进工作作风、密切联系群众的中央“八项规定”、省委20条和市委21条规定，大力弘扬求真务实之风，动真格整治“庸懒散奢贪”。切实精简会议，压缩文件，改进文风会风，提倡开短会，开有用的会，开解决问题的会，开高效务实的会；提倡讲短话，讲真话，讲心里话，讲有用的话，讲有建设性批评的话，不拉拉扯扯，不左顾右盼，不见风使舵，不避实就虚，不阳奉阴违，真正把心思和精力用在发展改革稳定上，用在为民办实事办好事解难忧上，用在立足本职工作争做更大贡献上。做到干部清正、政府清廉、政治清明。进一步强化抓落实的责任制，对重点工作、重点项目、招商引资、为民办实事事项等分解细化到各区各部门，做到工作有部署、有督促、有检查、有奖惩、有必要的事后评估，积极营造为人民、顾大局、干实事、能负责、有朝气、勇担当、出实效、守规矩的工作氛围。进一步强化政府系统的责任意识、机遇意识、创新意识和实干精神，不断增强政府工作人员履职尽责的能力和水平。四是切实加强廉政建设。坚决反对大手大脚、铺张浪费，从严控制“三公”经费和会议活动经费，加强公务接待管理，严格执行出国（境）和职务消费有关规定。认真落实廉政建设责任制，加强廉政风险防控，用制度“制权、管人、控钱”。对行政审批、招投标、土地出让、工程建设、政府采购、国有资产产权交易等重点领域和关键环节加强监管，对财政预决算、公共资源配置和重大项目建设强化审计。坚决依法查处违法违纪案件。

　　各位代表！时代赋予重托，实干铸就梦想。让我们在市委的坚强领导下，凝神聚力，务实创新，埋头苦干，努力完成今年的各项目标任务，为打造海南的“首善之城”而努力奋斗!
